# Transfer of nursing home residents to emergency departments: organizational differences between nursing homes with high vs. low transfer rates

**DOI:** 10.1002/nop2.68

**Published:** 2016-10-25

**Authors:** Marie Kirsebom, Mariann Hedström, Ulrika Pöder, Barbro Wadensten

**Affiliations:** ^1^ Department of Public Health and Caring SciencesUppsala UniversityUppsalaSweden

**Keywords:** advance care planning, avoidable hospitalization, geriatric nursing, hospital admission, nursing homes, organization and administration

## Abstract

**Aim:**

To explore possible factors in the organization of nursing homes that could be related to differences in the rate of transfer of residents from nursing homes to emergency department.

**Design:**

Explorative.

**Method:**

In a single municipality, qualitative and quantitative data were collected from documents and through semi‐structured interviews with 11 RNs from five nursing homes identified as having the highest vs. six identified as having the lowest transfer rates to emergency department. Data were analysed by non‐parametric tests and basic content analysis.

**Results:**

All nursing homes in the highest transfer rate group and one in the lowest transfer rate group were run by private for‐profit providers. Compared with the *low* group, the *high* group had fewer updated advance care plans and the RNs interviewed had less work experience in care of older people and less training in care of persons with dementia. There was no difference in nursing home size or staff/resident ratio. The RNs described similar possibilities to provide palliative care, medical equipment and perceived medical support from GPs.

## 1 Introduction

Several studies have revealed a high rate of hospitalization and emergency department (ED) use among older people in general and nursing home residents in particular, identifying various reasons for the transfers. The main medical reasons identified for the transfers are respiratory symptoms (Ackermann, Kemle, Vogel, & Griffin, 1998), suspected infections (Givens, Selby, Goldfeld, & Mitchell, 2012) and falls (Kihlgren, Wimo, & Mamhidir, 2014; Kirsebom, Hedström, Wadensten, & Pöder, 2014). Older persons with dementia have a frequent pattern of transfer because they receive more care in general (Callahan et al., 2012) and hospitalization at the end of life is more frequent when no hospice care is provided at the nursing home (Intrator, Castle, & Mor, 1999; Miller, Gozalo, & Mor, 2001). It is of importance that older persons receive high quality care, treatment for chronic conditions and avoid inappropriate hospitalizations which can be potentially harmful (Swedish National Board of Health and Welfare and Swedish Association of Local Authorities and Regions, 2011). For example, a visit to the ED may result in acute confusion and a decline in health (Kihlgren, Nilsson, Skovdahl, Palmblad, & Wimo, 2004; Shanley, Sutherland, Stott, Tumeth, & Whitmore, 2008).

## 2 Background

As described above, several studies have looked at the frequency of and reasons for transfers of nursing home residents to EDs. Most studies have discussed how to decrease the number of transfers that are not justified on medical grounds, meaning cases where the older person could have been satisfactory cared for at the nursing home with consultation from the GP. It has been shown that transfers from nursing homes to EDs could be avoided (Naylor, Kurtzman, & Pauly, 2009; Ouslander et al., 2010; Saliba et al., 2000), for example, if certain symptoms were treated in nursing homes to a greater extent and with the guidance of an advance care plan (ACP) (Young, Barhydt, Broderick, Colello, & Hannan, 2010). There has been less research on the organizational factors in nursing homes that could explain the rate of transfer to hospital, although some such factors have been identified. Nursing homes with special care units including more advanced technology and nursing homes with hospice care have lower hospitalization rates (Intrator, Zinn, & Mor, 2004; Intrator et al., 1999; Miller et al., 2001). It has also previously been reported that RNs feel supported in their decision not to transfer to ED if the nursing home has a palliative approach and documented medical care plans (ACPs) (Kirsebom, Wadensten, & Hedström, 2012). The RN staffing level has also been reported to be an important indicator of quality in hospice care (Canavan, Aldridge‐Carlson, Sipsma, & Bradley, 2013). Studies by Josefsson, Sonde, and Wahlin (2007) and Karlstedt, Wadensten, Fagerberg, and Pöder (2015) regarding education levels and competence among RNs working in care for older people in Sweden have shown that the majority lacked a bachelor's degree and few had specialist training. In one hospital setting, it was found that an increase in a nurse′s workload by one patient increased the risk of a patient dying within 30 days of admission by 7% and an increase in education was associated with a decrease in this risk by 7% (Aiken et al., 2014). The presence of more physicians (Intrator et al., 1999; Lisk et al., 2012) and a larger proportion of RNs (Carter & Porell, 2003; Konetzka, Spector, & Limcangco, 2008) in nursing homes reduce the risk of hospital admissions. Moreover, nursing homes that are privately run and for‐profit are more likely to transfer residents to hospital (Carter & Porell, 2003, 2005; Dobalian, 2004; Kirsebom et al., 2014; Konetzka et al., 2008).

In a previous study by Kirsebom et al. (2014), great variation was shown in the proportion of transfer rates from the nursing homes to the ED and to better understand this variation, nursing homes with the highest versus lowest transfer rates to ED were identified in the study sample. In the present study, we chose to study these identified nursing homes with regard to organizational factors.

### 2.1 Aim

The aim of the present study was to explore possible factors in the organization of nursing homes that could be related to differences in the rate of transfer of residents from nursing homes to EDs.

## 3 The study

### 3.1 Design

An explorative design, using both qualitative and quantitative methods (Malterud, 2001) was employed to increase the likelihood of multifaceted results.

### 3.2 Method

#### 3.2.1 Setting

In Sweden, healthcare is a public responsibility financed primarily through taxation (Swedish National Board of Health and Welfare and Swedish Association of Local Authorities and Regions, 2010). The municipalities are responsible for long‐term care and services for older persons. The care for the older person has been contracted out according to law (SFS 2007:1091) and facilities are run by both private for‐profit providers and public/private non‐profit providers. The county councils are responsible for hospitals, primary healthcare and outpatient care (SFS 2001:453). The policy in Sweden favours older persons remaining in their ordinary homes as long as possible (Ministry of Health and Social Affairs, 2007). Therefore, many older persons are approximately 80 years of age when they move to a nursing home. People in nursing homes have extensive health and social care needs around the clock (Swedish National Board of Health and Welfare and Swedish Association of Local Authorities and Regions, 2013). According to Swedish National Board of Health and Welfare (2005) 25% of the beds in nursing homes are specialized in care of older persons with dementia but significantly higher proportion, 65%, is estimated to have a diagnose with dementia (Nordberg et al., 2007).

The study took place in a metropolitan area in Sweden (population 200,000). At the time of the study, there were one ED and 32 nursing homes in the area, 23 run by private for‐profit providers, seven run by public providers and two by private non‐profit providers. RNs in the nursing homes were on duty from 7 am ‐ 4 pm on weekdays and responsible for nursing home residents’ medical and nursing care. A general practitioner (GP) visited on rounds and was available during the daytime for telephone consultations. During the evenings, nights and weekends, on‐call RNs and GPs were responsible for emergency calls and visits. The on‐call RNs could confer by telephone or go to the nursing home to read the medical records and examine the resident before deciding whether the resident needed to be assessed by a physician.

According to Swedish legislation (SFS 2008:355), both GPs and RNs are required to keep medical records. The physicians are responsible for ACPs, which is synonymous with a medical care plan that should be updated at least once a year. The ACP is created during a meeting with the resident, family members, the GP, the RN and should include the most important information on each resident's health status and what treatment and care each resident prefers in case his/her health should deteriorate. The ACP could help the GP and RN provide care that is in accordance with the resident's and family members’ wishes as well as with the practice of person‐centred care (McCormack et al., 2010). RNs are responsible for nursing care plans, which describe patients’ care needs, nursing interventions, goals and outcomes.

#### 3.2.2 Sample

In a previous study by Kirsebom et al. (2014), the frequency of and reasons for transfer from all 32 nursing homes in a Swedish municipality to the ED were investigated. Included were residents living in a nursing home age 65 and older, with healthcare records including documented transfers to the ED during a 9‐month period in 2010. Data were collected by structured reviews of the electronic healthcare records. In that study, the proportion of transfers to the ED during the study period varied between 0‐1.03 transfers/bed. Based on these results, the nursing homes specialized in somatic care (the nursing homes specialized in psychiatric care were not included in this study) with the highest versus lowest frequency of such transfers were identified. Gaps between groups of nursing homes with low, median and high transfer ratios were detected and these gaps became the cut off for the selected groups. Hence, the five nursing homes with the highest transfer rates to the ED, that is, number of transfers to the ED/number of beds (range 0.66‐1.03) and the six nursing homes with the lowest rates (range 0.00–0.26) were chosen for this study. In this study, we chose to study these nursing homes with regard to known influential organizational factors. Of the 20 RNs employed at these 11 nursing homes, the RN with the longest work experience at each workplace was selected for an interview, because these 11 RNs were expected to be able to provide more information with regard to the study purpose. All of the RNs invited agreed to participate.

#### 3.2.3 Data collection

The data for this study were collected from November 2011–March 2012. The documentary data were collected from the municipal official reports, websites and healthcare records. The quantitative data collected covered the following topics, previously referred to as organizational factors (Carter & Porell, 2003; Intrator et al., 1999; Kirsebom et al., 2012; Konetzka et al., 2008; Miller et al., 2001; Young et al., 2010): (i) staffing; (ii) care philosophy (yes/no); (ii) existence of ACPs updated within the previous year; (iii) nursing care plans; (iv) the RNs’ education and years of experience; (v) and the medical equipment in the nursing homes. Data on staffing were obtained from an official report by Engqvist and Öhman (2011).

All of the nursing homes’ websites, where the facilities are described, were read to establish whether they expressed some sort of care philosophy or theory. Data on number of ACPs and nursing care plans were collected during on‐site visits and registered during a meeting with an RN at each nursing home. The RNs reviewed the records and the researcher recorded the date of latest ACP and presence of a nursing care plan on a study‐specific checklist. The researcher was positioned in the room so that neither the computer screen nor the healthcare records could be read. Data on the participating RNs’ education, years of experience and the medical equipment in the nursing homes were collected during the interviews described below.

The primary method to collect qualitative data is individual interviewing (Polit & Beck, 2008). Semi‐structured interviews were performed by the first author, a doctoral student with several years of experience as an RN in care for older people. A pilot interview was carried out prior to the interviews to test the interview guide. This interview was not included in the analysis. The semi‐structured interviews covered the nurses’ experiences of the following topics: planning and organization of nursing care in the nursing home, palliative care, medical support and care philosophy in the nursing home. The interviews were conducted in a quiet room in the nursing home at a time chosen by the respective RNs. The interviews, which lasted about 40 minutes, were digitally recorded and transcribed verbatim.

### 3.3 Analysis

The quantitative data were presented using descriptive statistics and analysed with the statistical program IBM SPSS statistics version 20 using non‐parametric tests (IBM Corp, 2010). For comparisons between the two groups (high vs. low transfer rate), chi‐square analysis was used to compare dichotomous variables and Mann–Whitney U‐test (two‐tailed) was used to compare continuous variables.

The interviews were analysed deductively by applying the interview themes in the analysis to determine their manifest content, which describes the obvious visible components of a text, using a basic content analysis (Weber, 1990). First, the transcripts were read while listening to the tape‐recorded interviews to check the accuracy of the text and to get an initial impression of the interview. The whole interview transcript was then re‐read and recording units (words or sentences that answered to the research topics) were identified. The recording units were then categorized and scrutinized for differences in content between the two groups. The initial analysis was carried out by three of the authors and validated by one author. Examples of recording units are presented in Table 1.

**Table 1:** Example of recording units and categories

Recording units	Categories
We begin already during the first meeting with the older person and his/her relatives. This varies with the individual, but we often talk about it then. Talk about what kind of help we can provide here and how. We usually don't do intravenous drip, but we can give pain relief medications and sedatives and the care we are able to provide here. So they know what to expect.	Organization
Of course the most important thing is that they don't feel any pain, that they don't have trouble breathing and that, well, that we don't push them too much to eat and eat.	Palliative care
It is round once a week and the GP pays a visit to those who needs to have a visit, we usually have an ACP just in order to get through them all. Otherwise, between 8‐16 you can reach the GP via fax or phone.	Medical support from GPs
I think it's really important that we listen to them, listen to find out what each of them wants help with, I try to tailor things to each individual.	Care philosophy

### 3.4 Ethics

According to national directives, formal approval from an ethics committee was not required (Swedish Code of Statutes, SFS 2003:460). The ethical requirements laid down in the Helsinki Declaration were followed (WMA Declaration of Helsinki, 2013). The managers of the selected nursing homes were first contacted and asked if the nursing home could participate in the study. The RNs were contacted prior to the data collection and informed verbally and in writing about the study, that participation was voluntary and that they had the right to discontinue at any time without any explanation or consequences to themselves. Written informed consent was obtained from all participants. The primary ethical consideration in this study was to not expose the identity of the residents, RNs or nursing homes. Efforts were made to ensure that the researcher could not see the healthcare records or computer screen during data collection about presence of ACP and nursing care plans. For this reason, the RNs at the nursing homes reviewed the records and gave the researcher the data. All information has been treated confidentially. The procedure is consistent with the ethical rules established in Sweden (Codex 2015).

## 4 Results

Results from the statistical tests showed that more nursing homes in the *high* than in the *low* group were run by a private for‐profit provider; no other differences were found regarding organization of care (Tables 2 and 3). More nurses in the *low* than in the *high* group had training in care for the older people with dementia. RNs in the *low* group had worked more years in care for older people than those in the *high* group; no other differences were found regarding the interviewed RNs’ work experiences and education (Tables 2 and 3). Over the course of one year, nursing homes in the high group had fewer updated ACPs than did nursing homes in the low group. There was no difference with regard to existing nursing care plans (Table 3).

**Table 2:** Nursing homes with high vs. low transfer rates to the ED – presentation of variables analysed with Mann–Whitney analysis

	Transfer rates to the ED		*z*;* p*
	High, *n* = 5	Low, *n *=* *6	
	Min‐max/M; Md		
Characteristics of nursing homes			
Number of beds	20–80/44; 42	22–51/34; 29.5	0.6; 0.52
RNs ratio/resident	0.05–0.19/0.11; 0.09	0.08–0.24/0.14; 0.14	0.7; 0.46
Assistant nurses ratio/resident	0.43–2.47/1.52; 1.73	0.92–1.92/1.58; 1.64	0.4; 0.71
Interviewed RNs’ work experience			
Years as RN	2.5–26/10.3;7	12–30/20.5;20	1.8; 0.07
Years in care for the older people	2–5.5/3.2;2.5	11–30/16.3;14.5	2.8; <0.01

**Table 3:** Nursing homes with high vs. low transfer rates to the ED – presentation of variables analysed with chi‐square analysis

	Transfer rates to the ED		χ^2^; *p*
	High, *n *=* *5	Low, *n *=* *6	
	*n*		
Provider of care			
Private for‐profit	5	1	7.6; 0.01
Public and private non‐profit	0	5	
Care philosophy			
Care philosophy on website	5	6	n.a.^a^
RNs’ knowledge about the care philosophy	3	3	0.1;0.7
Interviewed RNs’ education			
Specialist education	1	3	1.0; 0.3
Dementia education	1	6	7.5; 0.01
Medical equipment at NH			
Peripheral vein catheter	5	6	n.a.^a^
Infusion equipment	5	6	n.a.^a^
Oxygen equipment	0	0	n.a.^a^
Blood pressure monitor	5	6	n.a.^a^
ECG	0	1	0.9; 0.33
ACPs and nursing care plans	*n*/total number of residents		
ACPs updated during past year	103/219	122/191	12.6; 0.00
Existing nursing care plans	186/219	151/191	1.5; 0.22

a Statistics not possible to calculate due to no variability.

The results from the interviews are presented in the following categories: organization of care, medical support from GP's, palliative care and care philosophy.

### 4.1 Organization of care

In all nursing homes in the high group, the RNs had delegated the task of drug administration to nursing assistants. In one of the nursing homes in the low group, the RNs reported handling all drug administration. In the other nursing homes, the RNs had delegated the task to nursing assistants.

At some of the nursing homes in the low group, the staff could phone the RNs during evenings and weekends when the RNs were off duty to consult on a resident before calling the on‐call RNs. They described how this improved the outcome for the resident and how transfers to the ED could be avoided. Further, one RN in the *low* group stated that because they are a small nursing home, they had time for their residents as well as for the ACPs and nursing care plans. One RN in the *high* group said they prioritized the frailest residents receiving their ACP first. Some RNs felt it was problematic when the on‐call RN did not follow the resident's ACP in an acute situation. The on‐call RNs often lacked time to read the medical record and were not familiar with the patient situation, which led to perceived unnecessary transfer to the ED. In both groups, the RNs described there not being any consensus regarding how documentation in the ACPs and nursing care plans should be written and stored.

### 4.2 Medical support from GPs

In general, one or two GPs were linked to each nursing home, with rounds occurring once or twice a week, depending on the number of residents in the nursing home. One RN from the high group and one from the *low* group had experienced fewer visits from GPs during the past year. According to these two RNs, there was a lack of continuity and there was not enough time spent to establish new or to revise old ACPs. The other RNs were satisfied with the medical support from the GP and mentioned that their GP would make an extra visit when needed.

### 4.3 Palliative care

All RNs reported having local guidelines for palliative care and that they adhered to them. Preferences for end of life should be discussed with the GP, the resident's immediate family and if possible the resident before the onset of acute illness. They described that palliative nursing care planning and an informed and involved immediate family are necessary to avoid acute transfers to the ED. All RNs talked about the importance of providing good palliative care. The resident should not experience any pain, anxiety or respiratory difficulties. Hence, medications for these symptoms should be available for each resident in the nursing home. In this context, they also stressed the importance of having an up‐to‐date ACP for all residents so that measures to be taken when a resident's health deteriorates were already decided.

In the *high* group, one RN mentioned that most residents prefer to be transferred to ED in the event of deteriorated health. In the *low* group, one RN mentioned that most residents wish to stay at the nursing home instead of receiving acute hospital care. At one nursing home in the *low* group, all RNs and GPs had received special training in palliative medical care. It was reported that this training had resulted in more consensus and that GPs prescribed a standard set of medications when a resident was in the palliative phase.

### 4.4 Care philosophy

Not all RNs were familiar with the care philosophy of their nursing home (Table 3). The RNs talked in general terms about the residents’ self‐determination and respect for the individual and reported thinking about their own preferences in the event that one of their own relatives should need care at a nursing home.

## 5 Discussion

Deciding the optimal level of care for each individual is difficult but essential. Extensive research have identified severe risks for older patients in being transferred to ED (Ong, Sabanathan, Potter, & Myint, 2011; Ouslander et al., 2010; Payne, Hardey, & Coleman, 2000), although ED visits are justified for conditions that cannot be sufficiently treated at the nursing home. In our study, nursing homes with low transfer rates have updated ACPs to a higher degree. It might be argued that this finding indicate that residents medical conditions are acknowledged rather than neglected, which otherwise could be a plausible explanation of low transfer rates.

All nursing homes with *high* transfer rates to ED were run by private, for‐profit providers, in contrast to the *low* group where only one was run by a for‐profit provider. Studies have demonstrated such differences between types of care providers (Carter & Porell, 2005; Dobalian, 2004; Kirsebom et al., 2014; Konetzka et al., 2008). These differences have not yet been explained sufficiently. In Sweden, privately run nursing homes are a relatively new phenomenon. It is possible that differences in transfer rates could partly be explained by differences in work experience among nurses, as nurses in private settings in Sweden are possibly more likely to have less experience. Another factor that differed between the *low* vs. *high* facilities was the RNs’ educational level, where RNs in the *low* group all had training in care of older people with dementia. The studies of Josefsson et al. (2007) and Karlstedt et al. (2015) regarding education levels and competence among RNs working in Sweden showed that the majority lacked a Bachelor's degree and few had specialist training. Nursing homes employing nurse practitioners with a Master's degree have been shown to have lower hospitalization rates (Intrator et al., 2004). Hence, increasing the competence of staff and RNs would appear to be one way of reducing transfers to the ED and hospitalization among nursing home residents.

The National Board of Health and Welfare has stressed the importance of increasing the formal competence level among RNs working with older people in Sweden (The National Board of Health and Welfare, 2012). The RNs could influence this situation by negotiating with their managers about continued education. RNs play an important role in pointing out what factors need to be improved and in arguing for changes to ensure that older persons in nursing homes receive good care.

All of the studied nursing homes had RNs on site only during the daytime on weekdays; at other times the unlicensed staff on duty could contact an on‐call RN. It can be discussed whether transfers would decrease if RNs were on duty even during evening shifts and weekends. There is some support for the notion that increasing RN staffing could lower transfer rates (Carter & Porell, 2005; Konetzka et al., 2008). The RN staffing level has also been reported to be an important indicator of quality in hospice care (Canavan et al., 2013) and in hospitals (Aiken et al., 2014). However, no difference in staff ratio was detected in the present study (McCormack et al., 2010). Heavy workload has been highlighted as the main cause of stress among RNs in nursing homes. We can only speculate that reducing stress levels and having time for the patient as well as making clinical assessments at an early stage in cases of deteriorating health could reduce the need of ED visits.

There were no differences between the high and low groups regarding medical equipment and experiences of support from the GP. None of the nursing homes had a great deal of medical equipment and it can be discussed whether having more medical equipment available could be one way of reducing avoidable transfers to ED. Zimmer, Eggert, Treat, and Brodows (1988) emphasized that if transfers are prevented by applying acute‐care medical healthcare in nursing home facilities, cost savings may occur. However, use of most medical equipment requires RN‐level competence and thus having and using additional equipment also entails RNs working evening and weekend shifts.

Nursing homes in the *high* group had fewer updated ACPs within the past year compared with the *low* group, a finding that is in accordance with previous discussions (Ong et al., 2011). Taken together, the findings suggest that updated ACPs seem to serve the purpose of guiding the decision‐making process when determining the level of care and avoiding inappropriate hospital admissions. ACPs also constitute a tool to promote person‐centred care. In interviews with GPs, they reported that planning ACPs and competence among RNs were crucial to the quality of care (Kirsebom, Hedström, Pöder, & Wadensten, 2016). These factors require that RNs have the ability to influence the situation by informing the GP about which older persons’ ACPs need to be up‐dated, given that the RNs have closer contact with the older persons and can see changes in their health status. In future research, it would be interesting to study organization of care, out‐of‐hours services and access to medical equipment in relation to the multi‐ill elderly population in nursing homes. RNs are on duty only during the day and residents are in need of 24‐hour services.

### 5.1 Limitations

The nursing homes with high and low transfer rates were identified in a retrospective data collection from 2010, whereas the data in this study were collected from November 2011–March 2012. Thus, organizational factors might have changed during the interval, although no nursing home had changed provider during this time. The efforts made to minimize this risk were to interview RNs who had been working at the respective nursing homes longer than 2 years. In addition, no data concerning the residents’ health status were included in this study. Thus, conclusions as to whether the transfers were justified or not cannot be drawn. The descriptive correlational approach in this study and the small sample hampers the alternatives for statistical analysis methods and control of possible confounding factors. The results merely describe relationships among variables, no causal relationships can be inferred from the findings and the results cannot be generalized (Polit & Beck, 2008). However, findings from exploratory studies such as this one do generate hypotheses that can be tested in the future.

The qualitative data were collected by semi‐structured interviews and had a descriptive approach. In retrospect, the data could have benefitted from a more open approach, to allow for further abstraction. The interviews were conducted by the first author, who had limited experience of qualitative interviewing but many years of experience from care for the older people in both public provider and private for‐profit provider settings, promoting sensitivity to the subject. The other researchers were all experienced qualitative researchers. The authors’ varied perspectives can be seen as strength as it may have improved the clarity of the study.

One strength of the present study is the explorative approach that includes both qualitative and quantitative data, which promotes better understanding of the phenomenon under study. Selection of factors to be studied was guided by previous research but interviews also resulted in new factors: staff members’ possibility to contact the nursing home's RNs during evenings and weekends before calling the on‐call RNs and the delegation of drug administration to nursing assistants. It would be interesting to investigate these factors in future studies. Other important research topics are on‐call RNs’ views on what would help them in making assessments and decisions as well as staff levels and the possible contribution of having RNs on duty around the clock. Furthermore, the context and the research process were described, enabling the reader to determine the trustworthiness and the transferability of the findings to similar settings.

## 6 Conclusion

Taken together, the present findings indicate that organizational factors could be related to differences in transfer rates between nursing homes. Our data reveal that nursing homes identified with the highest transfer rates to ED were run by for‐profit providers to a higher extent and had updated ACPs for their residents to a lower extent than did nursing homes with the lowest transfer rates. Further, RNs’ level of competence may be related to transfer rates. Improved use of ACPs in nursing homes needs to be in focus if the care provided is to conform to the wishes of residents and their families. Better adherence to ACPs could enhance the care and outcome for residents, thereby possibly reducing transfers to the ED. This needs to be further explored.

## Funding

Financially supported by Uppsala Municipality.

## Conflict of interest

No conflict of interest has been declared by the authors.

## Author contributions

All authors have agreed on the final version and meet the following criteria (recommended by the ICMJE (http://www.icmje.org/recommendations/):

- substantial contributions to conception and design, acquisition of data, or analysis and interpretation of data;
- drafting the article or revising it critically for important intellectual content.
